# MicroRNA-100 suppresses human osteosarcoma cell proliferation and chemo-resistance via ZNRF2

**DOI:** 10.18632/oncotarget.16149

**Published:** 2017-03-13

**Authors:** Qiang Xiao, Yu Yang, Qing An, Yong Qi

**Affiliations:** ^1^ Department of Hand Surgery, The First Affiliated Hospital of Liaoning Medical University, Jinzhou, 121001, China

**Keywords:** osteosarcoma (OS), ZNRF2, miR-100, cancer growth, chemo-resistance

## Abstract

Osteosarcoma (OS) is a prevalent cancer worldwide. MicroRNAs (miRNAs) play critical roles in the growth, invasion and carcinogenesis of OS, whereas the underlying mechanisms remain ill-defined. Here, we addressed these questions. We detected significantly higher levels of ZNRF2, a ubiquitin ligase of the RING superfamily, and significantly lower levels of miR-100 in the OS specimens, compared to the paired normal bone tissues. The levels of ZNRF2 and miR-100 inversely correlated in the OS specimens. In addition, low miR-100 levels are associated with poor prognosis of the OS patients. Either ZNRF2 overexpression or miR-100 depletion increased *in vitro* OS cell growth and improved cell survival at the presence of Doxorubicin. Mechanistically, with the help of bioinformatics analysis and luciferase-reporter assay, we found that miR-100 might bind to the 3’-UTR of ZNRF2 mRNA to prevent its protein translation. Thus, our data suggest that re-expression of miR-100 may inhibit OS cell growth and decrease OS cell chemo-resistance.

## INTRODUCTION

Osteosarcoma (OS) is the most prevalent malignant bone tumor, and is characterized by malignant mesenchymal cells that produce either osteoid or immature bone. Untreated OS may undergo a relentless course with disease progression and leads to death within months [1]. Surgical removal of the primary OS is still the basic treatment, while chemotherapy can either be administered before or after surgery [2]. Although the advances in chemotherapy have substantially improved the long-term survival of patients with OS, some OS are not sensitive to chemotherapy, which highlights the importance of understanding the mechanisms underlying the chemo-resistance of OS cells [3].

Doxorubicin (DOX), also called Adriamycin, is the first-line drug for OS chemotherapy. Besides well-known for its cardiotoxicity, DOX also suffers from the fact of being resistant by some OS cells [2]. Thus, it is important to figure out the mechanisms underlying the chemo-resistance of OS cells.

Protein synthesis requires participation of digested amino acids regulated by a molecule called mammalian target of rapamycin (mTor), a conserved serine/threonine protein kinase from the phosphatidylinositol 3-kinase -related kinase family [4–7]. The aberrant expression of mTor has been shown to be essential for the tumorigenesis of the majority of all cancers [8–11], including OS [12]. Recently, the membrane-associated E3 ubiquitin ligase ZNRF2 has been shown to be involved in the activation and regulation of mTor through protein interaction [13]. Moreover, ZNRF2 depletion has been shown to decrease cell size and cell proliferation [14]. However, the expression and regulation of ZNRF2 in OS is unknown.

MicroRNAs (miRNAs) are a group of small, non-coding RNAs [15–17]. Previous studies have demonstrated that aberrant miRNA expression affects normal bone homeostasis and metabolism, resulting in OS initiation and progression [18, 19]. Although a diverse role of miR-100 has been reported in different cancers [20–24], its effects on ZNRF2 expression specifically in OS has not been reported.

Here we reported significantly higher levels of ZNRF2 and significantly lower levels of miR-100 in the OS specimens, compared to the paired normal bone tissues. The levels of ZNRF2 and miR-100 inversely correlated in the OS specimens. Low miR-100 appeared to be associated with poor prognosis of the OS patients. Either ZNRF2 overexpression or miR-100 depletion increased *in vitro* OS cell growth and improved cell survival at the presence of Doxorubicin. Mechanistically, with the help of bioinformatics analysis and luciferase-reporter assay, we found that miR-100 might bind to the 3’-UTR of ZNRF2 mRNA to prevent its protein translation.

## RESULTS

### High levels of ZNRF2 and low levels of miR-100 are inversely correlated in OS

**Figure 1:**
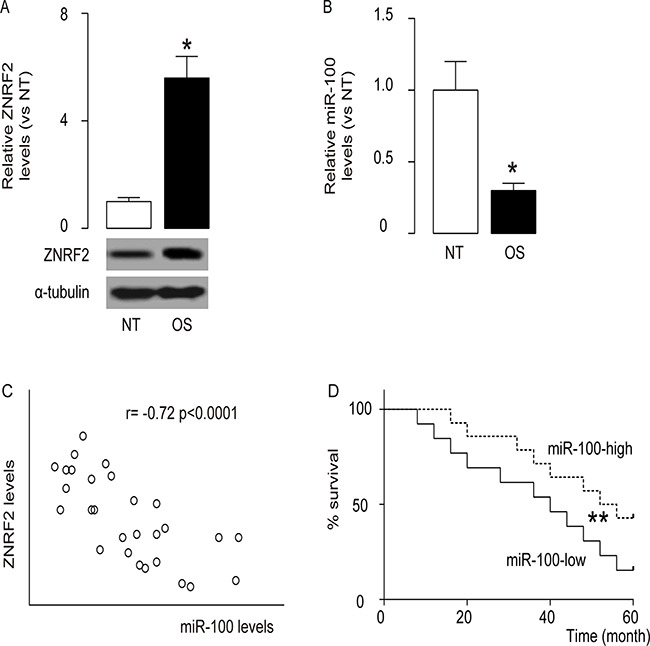
High levels of ZNRF2 and low levels of miR-100 are inversely correlated in OS ZNRF2 levels and miR-100 levels were examined in the resected OS specimens from 28 patients, and compared to the paired normal bone tissue (NT). **(A)** ZNRF2 by Western blot **(B)** miR-100 by RT-qPCR. **(C)** Correlation test between ZNRF2 and miR-100 levels in OS specimen (r= -0.72; p < 0.0001). **(D)** The survival of the 28 patients all diagnosed Stage IV were followed for 5 years. The median value of all 28 cases was chosen as the cutoff point for separating miR-100-high cases (n=14) from miR-100-low cases (n=14). Kaplan-Meier curves were performed. *p<0.05. **p<.01. N=28.

From the candidate ZNRF2-targeting miRNAs, we specifically found miR-100 as an interesting one, since in the resected 28 OS specimens along with the paired normal bone tissue (NT) (Table 1), we detected significant changes in miR-100 levels (Supplementary Table 1). Indeed, we not only detected significantly higher levels of ZNRF2 by Western blot (Figure 1A), but also detected significantly lower levels of miR-100 by RT-qPCR (Figure 1B) in the OS samples, compared to those in NT. Moreover, a significant inverse correlation was found between ZNRF2 and miR-100 levels in OS specimens (Figure 1C, r= -0.72; p < 0.0001). Next, we investigated whether the levels of miR-100 may correlate with 5-year survival of OS patients. The median value of miRNA the 28 cases was chosen as the cutoff point, after which Kaplan-Meier curves were performed, showing that miR-100-high OS patients had a significantly better survival (Figure 1D). Thus, decreased miR-100 in OS is associated with poor prognosis.

**Table 1:** Clinical-pathological characteristics

	Patients (n; %)	p
OS tissue/ Normal tumor-adjacent tissue (NT)	28 (100%)/28 (100%)	
Age (<60/≥60 years old)	21 (75%)/7 (25%)	0.45
Gender (male/female)	21 (75%)/7 (25%)	
Tumor site (bone)	28 (100%)	
Tumor grade (well or moderate/poor)	0 (0%)/18 (64%)/10 (36%)	0.02
Tumor stage (I/II/III/IV)	0 (0%)/6 (21%)/16 (56%)/6 (21%)	0.02
Lymph node metastasis (no/yes)	0 (0%)/28 (100%)	0.008
Distal metastasis at diagnosis (no/yes)	22 (79%)/6 (21%)	0.009

### Experimental changes of ZNRF2 levels in OS cells

**Figure 2:**
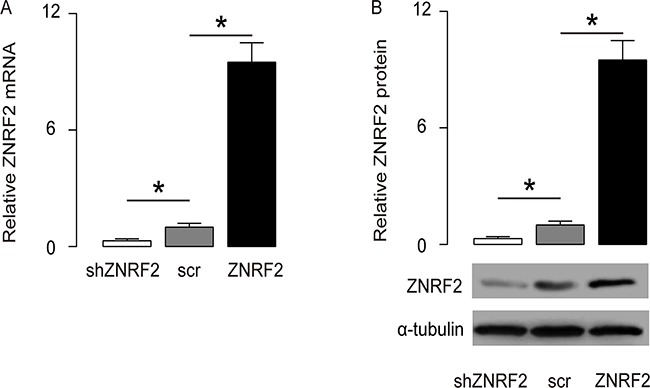
Modulation of ZNRF2 levels in OS cells U2OS cells were transfected with either a ZNRF2 overexpressing plasmid (ZNRF2), or a small short hairpin interfering RNA for ZNRF2 (shZNRF2). The U2OS cells were transfected with a scrambled sequence as a control (scr). These plasmids all carried a GFP reporter to allow determination of the transfection efficiency and purification of the transfected cells. **(A-B)** The modulation of ZNRF2 levels in these cells were confirmed by RT-qPCR **(A)**, and by Western blot **(B)**. *p<0.05. N=5.

In order to understand the molecular basis of these clinical findings, a human OS cell line U2OS was used in the current study. We transfected the U2OS cells with either a ZNRF2 overexpressing plasmid (ZNRF2), or a small short hairpin interfering RNA for ZNRF2 (shZNRF2). The U2OS cells were transfected with a scrambled sequence as a control (scr). These plasmids all carried a GFP reporter for determination of the transfection efficiency and for transfected cell purification. Next, alteration of ZNRF2 levels in these cells was confirmed by RT-qPCR (Figure 2A), and by Western blot (Figure 2B).

### ZNRF2 enhances OS cell growth and improves survival against DOX

**Figure 3:**
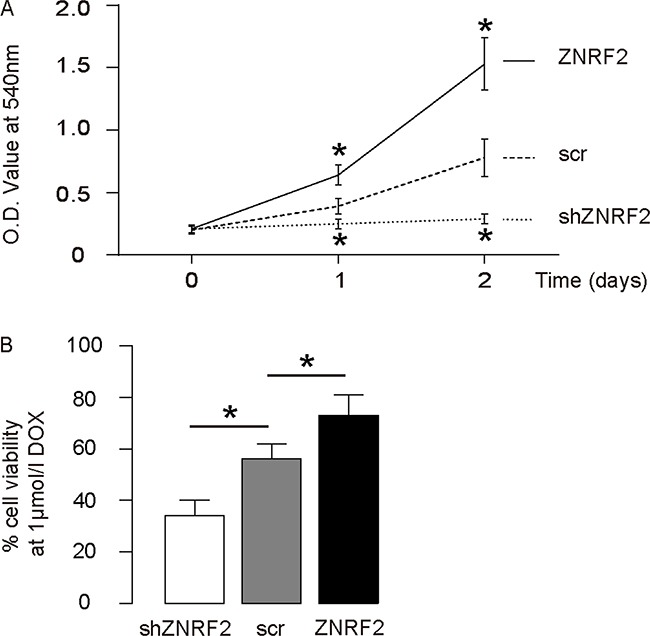
ZNRF2 enhances OS cell growth and improves survival against DOX **(A)** Cell growth of ZNRF2-modifed U2OS cells was examined in an MTT assay. **(B)** Cell survival of ZNRF2-modifed U2OS cells was examined in an CCK-8 assay at the presence of DOX. *p<0.05. N=5.

Next, the changes in cell growth in miR-100-modified cells were checked in an MTT assay. We found that overexpression of ZNRF2 in U2OS cells significantly increased cell growth, while ZNRF2 depletion significantly decreased cell growth (Figure 3A). The cell survival at the presence of DOX was then examined in an CCK-8 assay, showing that overexpression of ZNRF2 in U2OS cells significantly increased cell survival, while ZNRF2 depletion significantly decreased cell survival (Figure 3B). These data demonstrate that ZNRF2 may enhance OS cell growth and improve OS cell survival at DOX.

### Functional binding of 3’-UTR of ZNRF2 mRNA by miR-100

**Figure 4:**
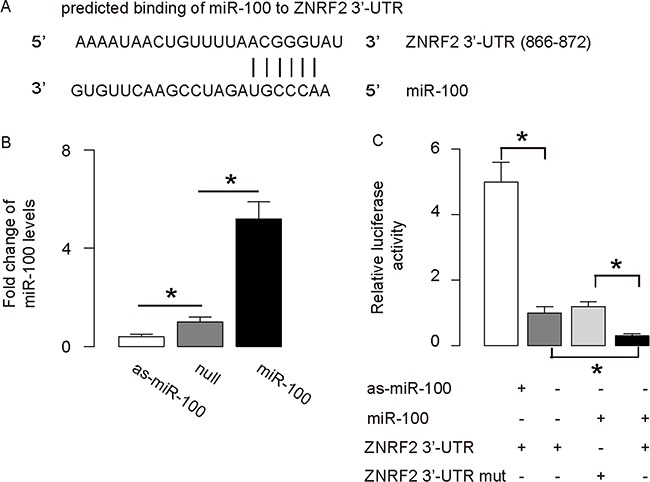
MiR-100 targets 3’-UTR of ZNRF2 to inhibit its expression **(A)** Bioinformatics analysis of ZNRF2 target sequence, which showed that miR-100 binds to 3’-UTR of ZNRF2 mRNA at 886^th^-872^th^ base site. **(B)** RT-qPCR for miR-100 in miR-100-modified U2OS cells. **(C)** The intact 3’-UTR of ZNRF2 mRNA (ZNRF2 3’-UTR), together with a 3’-UTR with mutant at miR-100-binding site of ZNRF2 mRNA (ZNRF2 3’-UTR mut), was then cloned into luciferase reporter plasmids, used for co-transfection with miR-100-modified plasmids. Luciferase activity was determined. *p<0.05. N=5.

Next, we performed bioinformatics analysis on the ZNRF2 targeting sequence for miRNA, which showed the miR-100-binding sites at 3’-UTR of ZNRF2 mRNA (Figure 4A). The plasmids that modifies miR-100 levels were prepared and validated (Figure 4B). The intact 3’-UTR of ZNRF2 mRNA (ZNRF2 3’-UTR), or a 3’-UTR with a mutant at miR-100-binding sites on ZNRF2 mRNA (ZNRF2 3’-UTR mut), was co-transfected with miR-100-modified plasmids in a dual luciferase reporter assay, showing that miR-100 specifically targets 3’-UTR of ZNRF2 mRNA to inhibit its translation in OS cells (Figure 4C).

### MiR-100 regulates OS cell growth and chemo-sensitivity through ZNRF2

**Figure 5:**
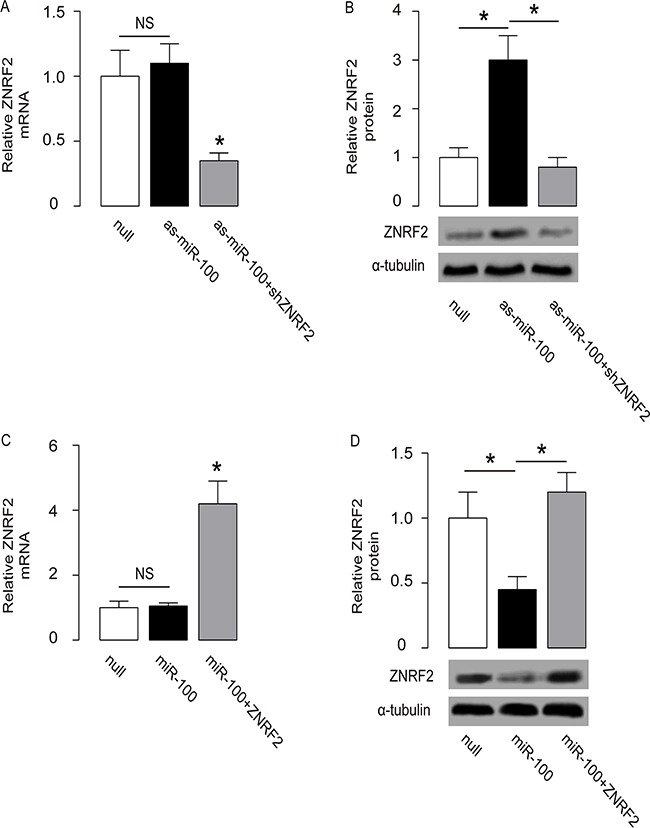
Co-transfection of OS cells **(A-B)** U2OS cells were co-transfected with as-miR-100 and shZNRF2, and the levels of ZNRF2 were examined by RT-qPCR **(A)** and by Western blot **(B)**. **(C-D)** U2OS cells were co-transfected with miR-100 and ZNRF2, and the levels of ZNRF2 were examined by RT-qPCR **(C)** and by Western blot **(D)**. *p<0.05. N=5.

**Figure 6:**
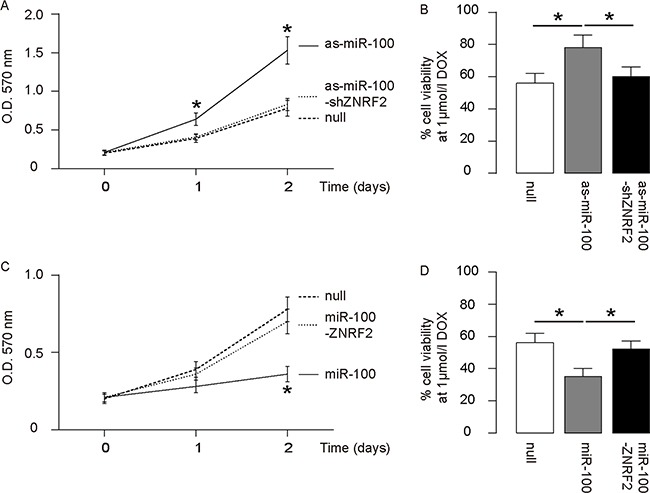
MiR-100 regulates OS cell growth and chemo-sensitivity through ZNRF2 **(A-B)** ZNRF2 suppression abolished the effects of as-miR-100 expression on cell growth **(A)** and chemo-sensitivity **(B)** in U2OS cells. **(C-D)** Augmentation of ZNRF2 abolished the effects of miR-100 expression on cell growth **(C)** and chemo-sensitivity **(D)** in U2OS cells. *p<0.05. N=5.

**Figure 7:**
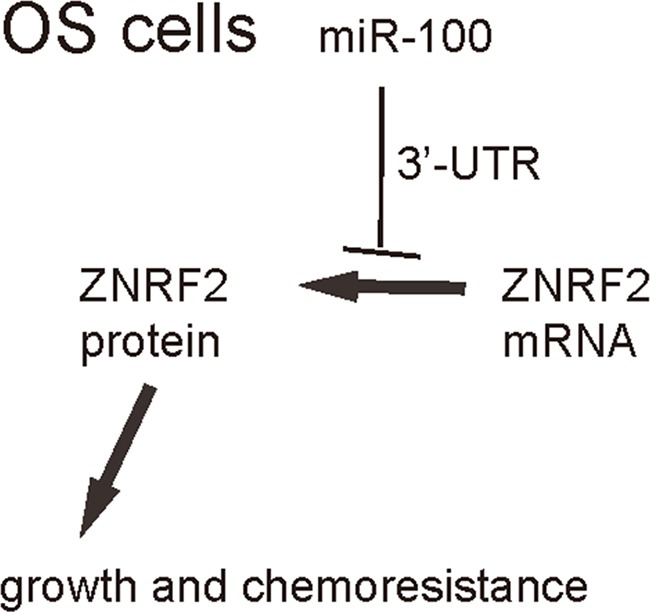
Schematic of the model MiR-100 inhibits OS cell growth and suppresses its chemo-sensitivity through ZNRF2.

We were thus prompted to evaluate whether miR-100 may regulate OS cell growth and chemo-sensitivity through ZNRF2. First, U2OS cells were co-transfected with as-miR-100 and shZNRF2. Specifically, the effects of as-miR-100 on ZNRF2 protein compromised the effects of shZNRF2 on ZNRF2 protein, which explained the findings in OS cells transfected with both as-miR-100 and shZNRF2 (Figure 5A–5B). Next, U2OS cells were co-transfected with miR-100 and ZNRF2 (Figure 5C–5D). We found that ZNRF2 suppression abolished the effects of as-miR-100 expression on cell growth (Figure 6A) and chemo-sensitivity (Figure 6B) in U2OS cells. Augmentation of ZNRF2 abolished the effects of miR-100 expression on cell growth (Figure 6C) and chemo-sensitivity (Figure 6D) in U2OS cells. These data suggest that miR-100 may suppress OS cell growth and chemo-resistance through ZNRF2 (Figure 7).

## DISCUSSION

ZNRF2 is a membrane-associated E3 ubiquitin ligase involved in the activation and regulation of mTor through protein interaction [13]. Hoxhaj et al. showed that release of ZNRF2 into the cytosol is mediated through Akt phosphorylation by Akt [14]. Membrane interaction between ZNRF2 and mTOR initiates the amino acid-stimulated translocation of mTORC1 to lysosomes, resulting in activation of mTOR signaling to augment cell growth [14]. Upon growth factor and amino acid stimulation, ZNRF2 is phosphorylated on Ser145 by mTORC1, which enhances vesicle-to-cytosol translocation of ZNRF2 as a negative feedback. Hence, ZNRF2 appeared to be a novel factor that may be involved in malignant cell growth [14]. Since the biological function of ZNRF2 was very recently discovered, its participation in the cancer growth, progress and metastasis remain largely unexplored.

Previous studies have confirmed the importance of miRNAs in the tumorigenesis, but a role of miR-100 in the tumorigenesis is just acknowledged very recently [20–24]. In this study, we aimed to study the molecular mechanisms that underlie the regulation of the tumor growth of OS by miR-100 and ZNRF2. We actually screened all miRNAs that target ZNRF2 using bioinformatics analyses, and picked up 5 candidates with the highest binding affinity. Then, we examined whether the expression levels of these 5 candidates may alter in OS specimens compared to NT. We specifically found that miR-100 was such a microRNA. To the best of our knowledge, the current study is the first study that showed a direct regulation of ZNRF2 by a miRNA, and specifically in OS. Our findings thus highlight miR-100/ZNRF2 axis as a novel therapeutic target for inhibiting the growth of OS and for suppression of their potential of chemo-resistance.

In future, the mechanisms underlying which the OS cells become re-sensitive to chemotherapeutic drugs may be analyzed, for example, changes in gene network that regulates cell apoptosis and autophagy.

The current study has several limitations. First, a larger number of clinical samples may further increase the strength of the study. Second, although we have checked cisplatin in our experimental settings and got similar results as DOX, additional chemotherapeutic drugs might be examined in miR-100-modified cells in future. Third, *in vivo* application of gene therapy in animal models of OS may provide *in vivo* data to better comprehend the effects of miR-100 re-expression in OS treatment. Finally, the influence of ZNRF2 expression on overall survival of examined patients may be examined to provide additional information.

To summarize, here we provide evidence to suggest miR-100/ZNRF2 axis as a novel therapeutic target for inhibiting the growth of OS and for suppression of their potential of chemo-resistance.

## MATERIALS AND METHODS

### Patient tissue specimens

A total of 28 resected OS specimens were compared with the paired normal bone tissue (NT) from the same patient (Table 1). All clinical samples were histologically examined and clinically diagnosed at the Liaoning Medical University from 2009 to 2010. Prior approval as well as the patient's consents were obtained from the Institutional Research Ethics Committee.

### Cell line culture and reagents

A human osteosarcoma cell lines U2OS was purchased from American Type Culture Collection (ATCC, Manassas, VA, USA), and cultured as previously described [25]. Doxorubicin (DOX; Sigma-Aldrich, St Louis, MO, USA) was applied to the cells at concentration of 1μmol/l in culture.

### Transfection

ZNRF2, scrambled control (scr) and short hairpin small interfering RNA for ZNRF2 (shZNRF2, sequence: 5’- TACGTCCAAGGTCGGGCAGGAAGA-3’) were cloned into pCMV-luciferase-2A-GFP vector (Clontech, Mountain View, CA, USA) to replace the GFP to generate pCMV-luciferase-2A-transgene. MiR-100, an antisense (as) of miR-100, and a null sequence (null) were similarly prepared. MiR-100 sequence is 5’- AACCCGUAGAUCCGAACUUGUG-3’, and as-miR-100 sequence is 5’-CACAAGUUCGGAUCUACGGGUU-3’. Sequencing was performed to confirm the correct orientation of the plasmids, which were then used to transfect the cells at a concentration of 100 nmol/l using Lipofectamine 2000 (Invitrogen, Carlsbad, CA, USA). The transfection efficiency was more than 95%, based on GFP expression.

### Prediction of miRNA targets and 3’-UTR luciferase-reporter assay

MiRNAs targets were predicted with the algorithm TargetScan, and the data were analyzed as previously described, using context+ scoring method [26]. The ZNRF2 3’-UTR reporter plasmid and the ZNRF2 3’-UTR reporter plasmid with a mutant at miR-100 binding site were both purchased from Creative Biogene (Shirley, NY, USA). The dual-luciferase reporter assay (Promega, Fitchburg, WI, USA) was performed 36 hours after transfection.

### Quantitative PCR (RT-qPCR)

Total RNA was extracted from cultured cells with miRNeasy mini kit (Qiagen, Hilden, Germany). Quantitative PCR was performed in duplicates, and the results were analyzed using 2^-△△Ct^ method. Values of mRNA were first normalized against α-tubulin, and then compared to the experimental controls, as described before [25].

### Western blot

The total cellular protein was extracted from the resected OS specimens or adjacent normal bone tissue (NT), or cultured cells. Primary antibodies for Western blot were anti-ZNRF2 and anti-α-tubulin (Cell Signaling, San Jose, CA, USA). HRP-conjugated anti-rabbit antibody (Jackson ImmunoResearch Labs, West Grove, PA, USA) was used as the secondary antibody. The ZNRF2 protein levels were first normalized to α-tubulin, and then normalized to experimental control by NIH ImageJ software (Bethesda, MA, USA), as described before [25].

### Cell viability assay

The CCK-8 detection kit (Sigma-Aldrich) was described before [27].

### Cell growth assay

A diphenyltetrazolium bromide (MTT) assay was described before [27].

### Statistical analysis

All statistical analyses were carried out using the SPSS 19 statistical software. The values are presented as mean ± SD and are considered significant if p < 0.05, analyzed with the method of one-way ANOVA with a Bonferroni correction. Fisher's Exact Test was applied to compare two groups. Kaplan-Meier analysis was used to analyze Patients’ survival.

## SUPPLEMENTARY MATERIALS FIGURES AND TABLES


