# Supplementary material for: MicroRNA-100 suppresses human osteosarcoma cell proliferation and chemo-resistance via ZNRF2
Source: Oncotarget. 2017 Mar 13;8(21):34678–86. doi: 10.18632/oncotarget.16149 (PMC5471002; doi:10.18632/oncotarget.16149)
Supplement: Supplementary file 1 [file oncotarget-08-34678-s001.pdf]

MicroRNA-100 suppresses human osteosarcoma cell proliferation and chemo-resistance via ZNRF2

SUPPLEMENTARY TABLE

Supplementary Table 1: Candidates ZNRF2-binding miRNAs

miRNA	Position in the UTR	seed match	context++ score	levels (OS vs NT)	p value
hsa-miR-107	1507-1514	8mer	-1.03	0.85±0.15	>0.05
hsa-miR-23c	504-511	8mer	-0.95	0.93±0.15	>0.05
hsa-miR-181a	544-551	8mer	-0.81	1.13±0.18	>0.05
hsa-miR-100	866-872	7mer-1A	-0.58	0.30±0.05	<0.05
hsa-miR-217	661-667	7mer-m8	-0.49	1.23±0.25	>0.05

For each predicted target of each miRNA, the sum of the context+ scores for the sites to that miRNA was calculated as the total context+ score. Predicted targets of each miRNA family are sorted by total context+ score. The representative miRNA is the miRNA in its family with the most favorable (lowest) total context+ score. From the examined 5 candidates, only significant alteration of miR-100 was detected in OS vs NT.
